# Peroxidase-like Activity of CeO_2_ Nanozymes: Particle Size and Chemical Environment Matter

**DOI:** 10.3390/molecules28093811

**Published:** 2023-04-29

**Authors:** Arina D. Filippova, Madina M. Sozarukova, Alexander E. Baranchikov, Sergey Yu. Kottsov, Kirill A. Cherednichenko, Vladimir K. Ivanov

**Affiliations:** 1 Kurnakov Institute of General and Inorganic Chemistry of the Russian Academy of Sciences, 119991 Moscow, Russia; 2 Department of Physical and Colloid Chemistry, Faculty of Chemical and Environmental Engineering, National University of Oil and Gas “Gubkin University”, 119991 Moscow, Russia

**Keywords:** cerium dioxide, colloids, surface, hydroxyl species, enzyme-like activity, buffer, size effect

## Abstract

The enzyme-like activity of metal oxide nanoparticles is governed by a number of factors, including their size, shape, surface chemistry and substrate affinity. For CeO_2_ nanoparticles, one of the most prominent inorganic nanozymes that have diverse enzymatic activities, the size effect remains poorly understood. The low-temperature hydrothermal treatment of ceric ammonium nitrate aqueous solutions made it possible to obtain CeO_2_ aqueous sols with different particle sizes (2.5, 2.8, 3.9 and 5.1 nm). The peroxidase-like activity of ceria nanoparticles was assessed using the chemiluminescent method in different biologically relevant buffer solutions with an identical pH value (phosphate buffer and Tris-HCl buffer, pH of 7.4). In the phosphate buffer, doubling CeO_2_ nanoparticles’ size resulted in a two-fold increase in their peroxidase-like activity. The opposite effect was observed for the enzymatic activity of CeO_2_ nanoparticles in the phosphate-free Tris-HCl buffer. The possible reasons for the differences in CeO_2_ enzyme-like activity are discussed.

## 1. Introduction

Nanocrystalline cerium dioxide is well known as a multifunctional catalyst [1], a UV-protective material [2,3,4] and a component for highly sensitive gas sensors [5,6,7]. One of the main factors determining the functional characteristics of ceria-based materials is the size of CeO_2_ particles. For instance, Wu et al. found that the rate of photoinduced decomposition of the herbicide, *N*-(phosphonomethyl)-glycine, decreases by a factor of 6 with an increase in the particle size of cerium dioxide from 2 to 5 nm [8]. Torrente-Murciano et al. demonstrated that doubling the particle size (from 5 to 10 nm) leads to a decrease in the catalytic activity of CeO_2_ in the reaction of naphthalene oxidation to CO_2_ by a factor of 2.5 [9]. It is important to note that the size effect is typical not only for ultrasmall particles of cerium dioxide (less than 10 nm), but also for larger particles (up to 50 nm). Lin et al. found that the rate of conversion of carbon dioxide to methane (at 548 K) decreases by a factor of 3 with an increase in the size of CeO_2_ particles from 32 to 50 nm [10]. The dependence of the catalytic activity of cerium dioxide on particle size is generally associated with a number of factors, including changes in the surface-to-volume ratio, band gap energy, surface chemistry and electronic structure [11].

In recent years, it has been found that cerium dioxide demonstrates exceptional biological activity, exhibits antibacterial [12,13,14] and antiviral properties [15,16], is characterised by low cytotoxicity [17,18,19], and can act as a UV- and radioprotective agent [20,21]. One of the key mechanisms of the biological action of CeO_2_ is associated with its ability to mimic the activity of a number of enzymes and exhibit peroxidase- [22], catalase- [23], oxidase- [24], superoxide dismutase- [25], lipoperoxidase-, phospholipoperoxidase- [26], phosphatase- [27], phospholipase- [28], photolipase- [29], haloperoxidase- [30] and urease-like activity [31]. In particular, Lang et al. demonstrated a direct correlation between the antiviral activity of cerium dioxide against human coronavirus OC43 and its haloperoxidase-like activity [30].

Since cerium dioxide is able to catalyse reactions involving enzyme substrates, it might be expected that the size of CeO_2_ particles will affect the rate of such reactions. There is, however, a scarcity of data in the literature on the effect of particle size on the enzyme-like activity of CeO_2_. Shlapa et al. showed that an increase in the size of CeO_2_ particles by a factor of ~2 (from 7 to 15 nm) leads to a decrease in the oxidase-like activity of cerium dioxide by a factor of 1.2 [24]. Henych et al. found that the phospholipase-like activity of CeO_2_ with a particle size of 5 nm is more than 30 times higher than the activity of 10 nm CeO_2_ particles [28]. To the best of the authors’ knowledge, there are virtually no data on the size effect on the peroxidase-like activity of CeO_2_. At the same time, hydrogen peroxide is the most important reactive oxygen species (ROS) that causes oxidative stress in living systems, and the peroxidase activity of enzymes and their mimetics is of paramount importance in biological processes [32].

As a rule, spectrophotometric methods, based on determining the concentration of coloured products of catalytic reactions, are used to determine the ROS-scavenging ability of materials. For this purpose, TMB (3,3′,5,5′-tetramethylbenzidine) assays are the most commonly used. Nevertheless, this approach has several limitations due to the complex mechanism of TMB oxidation that can proceed via either one-electron or two-electron pathways. Moreover, a charge-transfer complex between TMB and its diimine final product can form. All of these products are characterised by different absorption wavelengths, extinction coefficients and formation rate constants [33,34]. For a deeper insight into the chemical interactions of cerium dioxide with biological systems, however, selective methods for determining its enzyme-like activity are of primary importance, especially those that are specific to particular reactive oxygen species (e.g., OH·, HO_2_· and O_2_·^−^). In this context, fluorescent [22] or chemiluminescent [35] methods are considered to be more accurate and informative.

Special attention should be paid to the correct choice of the medium used for the analysis of enzyme-like activity, e.g., the choice of a physiologically relevant buffer solution [36]. It is generally accepted that it is the pH of the medium used that determines the mechanism of CeO_2_ interaction with hydrogen peroxide [37], while the presence of phosphate ions affects the activity of cerium dioxide, although not in a completely unambiguous way [38,39,40,41].

In this regard, the accurate analysis of the size effect on the enzyme-like activity of cerium dioxide requires the use of a single synthetic technique that will allow the production of CeO_2_ with different particle sizes under the same, or similar, conditions, as well as an analysis of its enzyme-like activity within a single analytical approach, taking into account the chemical environment of the nanoparticles. In the present work, a quantitative analysis of the size effect on the peroxidase-like activity of nanocrystalline cerium dioxide was carried out using the chemiluminescent method with two different buffer solutions (namely phosphate and Tris-HCl buffer solutions). These buffer solutions had the same pH (7.4) but differed in the presence or absence of phosphate ions.

## 2. Results

### 2.1. Synthesis of Aqueous Cerium Dioxide Sols with a Given Particle Size

Since cerium dioxide is characterised by low solubility in aqueous media and there is a weak dependence of particle size on synthesis temperature, the preparation of CeO_2_ sols with different sizes of nanoparticles is a complex problem. The approach used in the present study, based on the thermohydrolysis (95 °C) of ceric ammonium nitrate, makes it possible to obtain ultrasmall (up to 5 nm) CeO_2_ particles possessing high surface activity [42]. In this work, solutions of (NH_4_)_2_[Ce(NO_3_)_6_] with concentrations of 0.046, 0.092, 0.185, 0.277 and 0.370 M were used to obtain cerium dioxide with a high yield (Table 1). In the course of the preliminary experiments, it was found that the use of a ceric ammonium nitrate solution with a lower concentration (0.046 M) did not produce a CeO_2_ sol. The resulting solution did not demonstrate the Tyndall effect, which indicated the absence of CeO_2_ nanoparticles in the solution. As a result of the thermal treatment of the (NH_4_)_2_[Ce(NO_3_)_6_] solutions with concentrations of 0.092–0.370 M, a series of CeO_2_ sols with different particle sizes were obtained.

**Figure 1:**
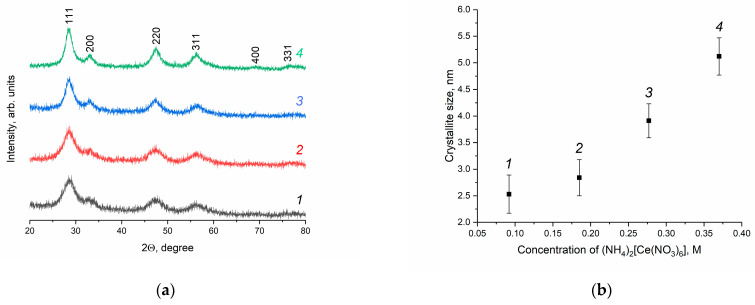
(**a**) XRD patterns of CeO_2_ nanopowders prepared upon the drying (50 °C) of aqueous ceria sols. (**b**) Dependence of CeO_2_ crystallite size on the concentration of ceric ammonium nitrate in the reaction mixture. Ceria sols were obtained from (1) 0.092 M, (2) 0.185 M, (3) 0.277 M and (4) 0.370 M solutions of (NH_4_)_2_[Ce(NO_3_)_6_].

The XRD patterns of the CeO_2_ sols, which were dried at a low temperature (50 °C), are shown in Figure 1. According to the data obtained, the phase composition of the solid residues corresponds with nanocrystalline cubic cerium dioxide (sp. gr. F*m*$\overset{-}{3}$*m*, PDF2 00-034-0394). As can be seen from Figure 1a, the peak width decreases with an increase in (NH_4_)_2_[Ce(NO_3_)_6_] concentration. CeO_2_ crystallite size was evaluated using the XRD data based on the Scherrer formula. According to Figure 1b, the size of cerium dioxide crystallites increases consistently, in the range of 2–5 nm, with the changes in the concentration of (NH_4_)_2_[Ce(NO_3_)_6_].

According to the generally accepted concepts of nucleation and crystal growth, the growth of solid-phase particles typically proceeds via the dissolution–crystallisation mechanism (Ostwald ripening). Conversely, for cerium dioxide and some other oxides, the mechanism of oriented attachment and growth of particles is usually observed [43,44], particularly under hydrothermal conditions [45]. Apparently, when taking into account the extremely low solubility of CeO_2_, the observed increase in the size of CeO_2_ particles with an increase in the initial concentration of (NH_4_)_2_[Ce(NO_3_)_6_] is due to the implementation of the nonclassical particle growth mechanism.

**Figure 2:**
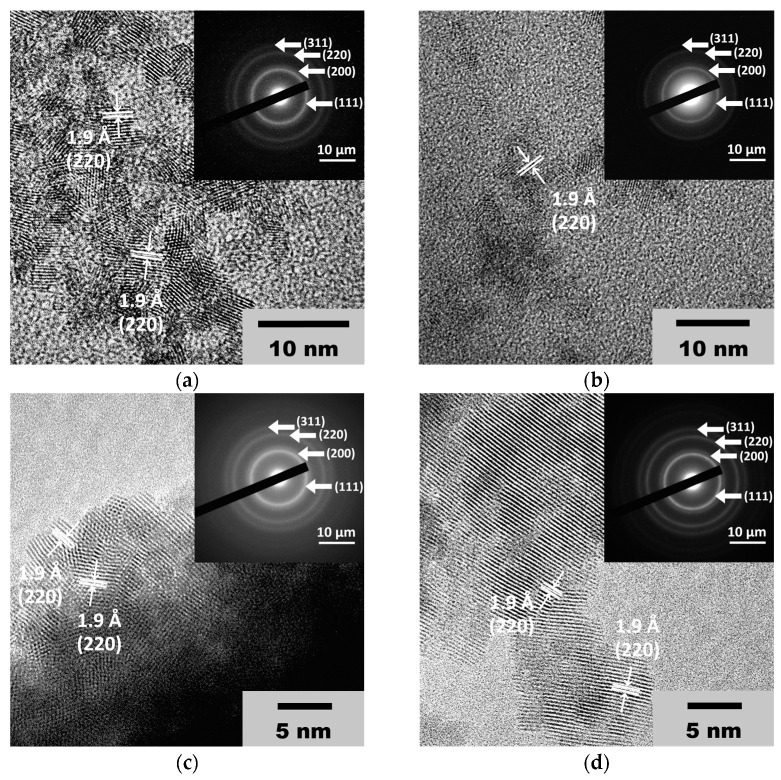
TEM images and SAED patterns of the CeO_2_ sols obtained from (**a**) 0.092 M, (**b**) 0.185 M, (**c**) 0.277 M and (**d**) 0.370 M aqueous solutions of (NH_4_)_2_[Ce(NO_3_)_6_].

The high-resolution transmission electron microscopic (HRTEM) images confirm the results of the X-ray diffraction. As can be seen from Figure 2 (see also ESI, Figure S1 (Supplementary Materials)), the CeO_2_ particle size is approximately 3 nm. The HRTEM images display interplanar distances of about 1.9 Å, which can be attributed to the (220) planes in the crystal lattice of CeO_2_ [46,47,48].

**Figure 3:**
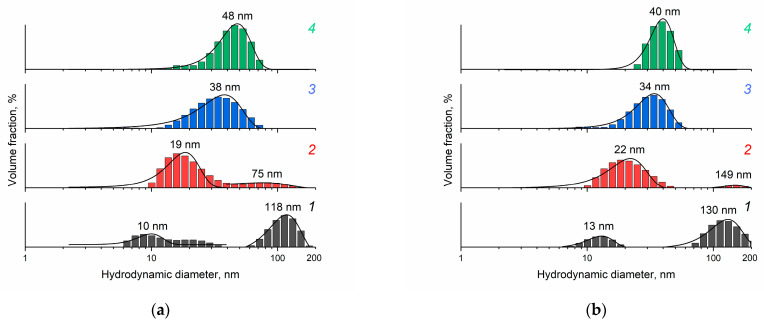
Hydrodynamic diameter distribution for CeO_2_ particles in aqueous ceria sols obtained from (1) 0.092 M, (2) 0.185 M, (3) 0.277 M and (4) 0.370 M (NH_4_)_2_[Ce(NO_3_)_6_] aqueous solutions: (**a**) after synthesis and (**b**) after three months of storage under ambient conditions. The pH value of all the sols is approximately 2.4.

The dynamic light-scattering technique allowed the study of the size distribution of aggregates of individual ceria nanoparticles in the sols (Figure 3). The CeO_2_ sol obtained from 0.092 M ceric ammonium nitrate solution is characterised by bimodal distribution of aggregates. As the concentration of (NH_4_)_2_[Ce(NO_3_)_6_] in the starting solution increases, a transition to monomodal distribution of aggregates in the sols is observed. At the same time, with an increase in (NH_4_)_2_[Ce(NO_3_)_6_] concentration, the size of aggregates increases. It should be noted that during the 3 months of storage under ambient conditions, the size of aggregates in the sol obtained from the solution with the lowest concentration of (NH_4_)_2_[Ce(NO_3_)_6_] increases by 30% (from 10 to 13 nm), and the size of agglomerates increases by 10% (from 120 to 130 nm), while the aggregate size of the sols obtained from the solutions with higher concentrations of ceric ammonium nitrate (0.185–0.37 M) changes by no more than 15% (Figure 3b).

**Figure 4:**
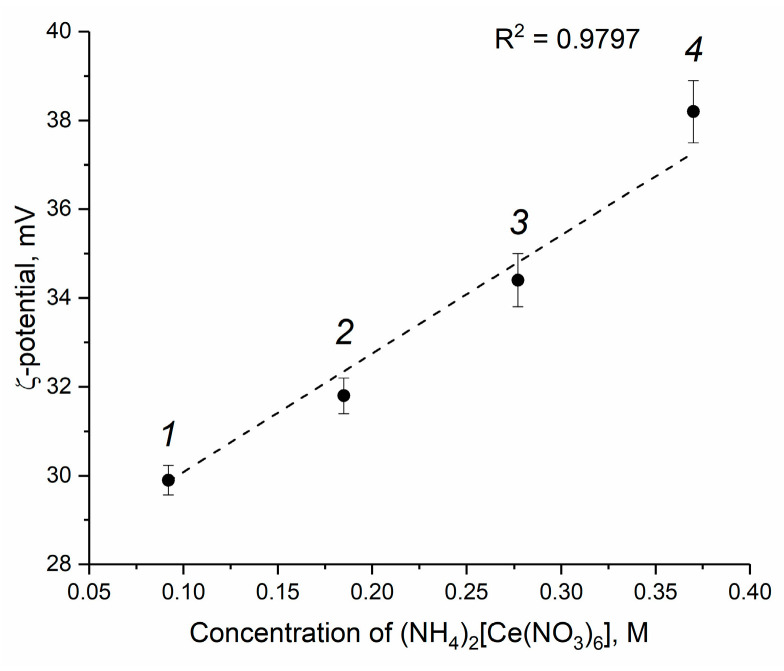
Dependence of the ζ-potential of CeO_2_ aqueous sols on the concentration of initial ceric ammonium nitrate solution. The sols were obtained from (1) 0.092 M, (2) 0.185 M, (3) 0.370 M and (4) 0.554 M solutions of (NH_4_)_2_[Ce(NO_3_)_6_]. The pH value of all the sols is approximately 2.4.

The results of the electrokinetic measurements are shown in Figure 4 and Figure S2. Thermohydrolysis of ceric ammonium nitrate yields an acidic environment; thus, CeO_2_ particles acquire a positive charge due to the protonation of surface hydroxyl groups [47]. The pH value of all of the sols obtained is approximately 2.4, so the values of the ζ-potentials are positive (Figure 4). As it follows from Figure 4, with an increase in the concentration of ceric ammonium nitrate in the starting solutions, the values of the ζ-potential of CeO_2_ nanoparticles increase from +29.9 to +38.2 mV. High ζ-potential values ensure long-term stability of CeO_2_ colloids, which demonstrate no signs of precipitation after three months of storage under ambient conditions (Figure 3).

**Figure 5:**
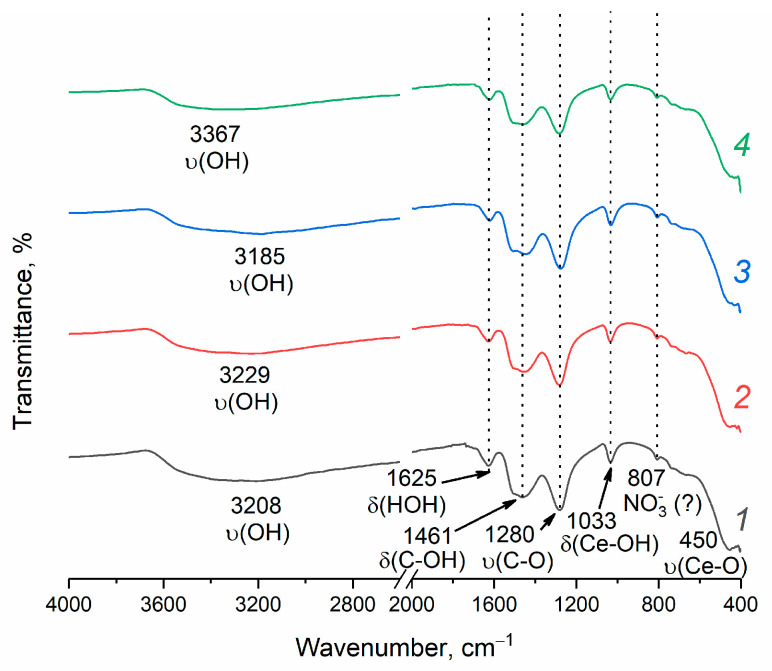
FTIR spectra of CeO_2_ nanopowders prepared by drying aqueous ceria sols obtained from (1) 0.092 M, (2) 0.185 M, (3) 0.277 M and (4) 0.370 M aqueous solutions of (NH_4_)_2_[Ce(NO_3_)_6_].

The IR spectroscopy (Figure 5) shows a broad absorption band in the 3400–3000 cm^−1^ region, which can be attributed to the stretching vibrations of physically adsorbed water [49]. The absorption band at 1625 cm^−1^ corresponds to the bending vibrations of molecular water [49]. The peaks at 1033 and 450 cm^−1^ can be attributed to the bending vibrations of the Ce–OH surface groups and to the stretching vibration of the Ce–O bond [50,51]. The absorption bands in the range of 1500–1300 cm^−1^ and at 1280 cm^−1^ correspond to the bending C–OH and stretching C–O vibrations of isopropanol traces [52]. The weak absorption band at 807 cm^−1^ can be attributed to the vibrations of nitrate species adsorbed on the oxide surface [53].

**Figure 6:**
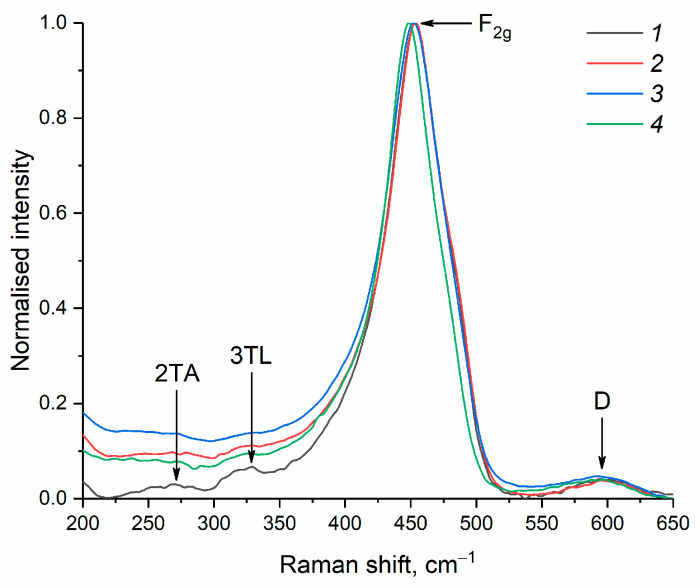
Raman spectra of CeO_2_ nanopowders prepared by drying aqueous ceria sols obtained from (1) 0.092 M, (2) 0.185 M, (3) 0.277 M and (4) 0.370 M solutions of (NH_4_)_2_[Ce(NO_3_)_6_].

The Raman spectra of the dried ceria sols are typical for nanocrystalline CeO_2_ (Figure 6) and show an intense F_2g_ mode peak at 453–448 cm^−1^, which is characteristic of a fluorite structure and is due to the symmetric stretching vibrations of the Ce-O bond [8]. A weak band at about 600 cm^−1^ can be attributed to the defect-induced (D) mode [54,55]. The band centred at ~270 cm^−1^ can be ascribed to the second-order transverse acoustic (2TA) mode [7]. The band at 330 cm^−1^ is characteristic of the 3TL mode of O-Ce-O vibrations [56].

The main physicochemical characteristics (particle sizes according to the XRD and DLS data, and ζ-potential values) of cerium dioxide colloidal solutions are presented in Table 2.

The specific surface area (SSA) of CeO_2_ was estimated from the particle sizes, determined according to the powder X-ray diffraction data and the literature data on the density of CeO_2_ (ρ = 7.215 g/cm^3^, PDF2 N°00-034-0394), according to Formula (1):

(1) $$SSA=6000/\left(D\cdot \rho \right)$$

where 6000 is the shape factor, D is the diameter of a spherical CeO_2_ particle, and ρ is the density of CeO_2_. The proportion of surface cerium atoms in spherical CeO_2_ particles (Ce_surf_) was also evaluated, according to a previously proposed method [57].

### 2.2. Enzyme-like Activity of CeO_2_ Sols towards H_2_O_2_ Decomposition

The chemiluminescent method used for the analysis of peroxidase-like activity is based on the interaction of a chemiluminescent probe molecule (luminol) with reactive oxygen species through the formation of monoprotonated 3-aminophthalic acid in an electronically excited state [58]. The luminescence intensity of the luminol oxidation product is proportional to the concentration of free radicals, which depends on the activity of the enzyme-like inorganic material. This method of analysis makes it possible to determine the enzyme-like activity of nanomaterials with high analytical precision [59,60,61,62,63,64,65].

**Figure 7:**
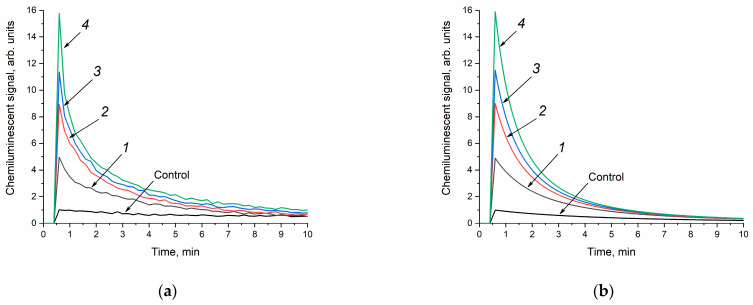
Luminol (50 μM) chemiluminescence kinetic curves in the presence of H_2_O_2_ (10 mM) and CeO_2_ sols (250 μM) in a phosphate-buffered solution (100 mM, pH = 7.4): the experimental data were obtained via direct chemiluminescent measurements (**a**), and the calculated curves were obtained through kinetic modelling of the experimental data (**b**). The aqueous ceria sols were obtained from (1) 0.092 M, (2) 0.185 M, (3) 0.277 M and (4) 0.370 M solutions of (NH_4_)_2_[Ce(NO_3_)_6_]. Control: luminol and H_2_O_2_ in a phosphate-buffered solution without CeO_2_. The chemiluminescence signal was recorded at 36 °C.

At the first stage, the analysis of the enzyme-like activity of cerium dioxide was carried out in a phosphate-buffered solution. The concentration of cerium dioxide in the reaction mixture was the same for all the sols and amounted to 250 μM. When the cerium dioxide sols were added to the reaction mixture containing a phosphate-buffered solution, luminol and hydrogen peroxide, an increase in the luminescence intensity of the luminol oxidation product was observed (Figure 7a). Thus, cerium dioxide catalysed the oxidation of luminol in the presence of H_2_O_2_; however, the appearance of the chemiluminescence curves differs from those obtained for horseradish peroxidase [58]. It is most likely that, in a phosphate-buffered solution, cerium dioxide exhibits prooxidant (more precisely, peroxidase-like) activity [66]. In this case, the chemiluminescence intensity increases with an increase in the size of CeO_2_ particles. A similar effect was observed for the different concentrations of the CeO_2_ sols at 500 μM and 6 mM.

The kinetic parameters of the luminol oxidation reactions with hydrogen peroxide in the presence of CeO_2_ were mathematically modelled (Figure 7b). At the first stage, the interaction of cerium dioxide with H_2_O_2_ with the formation of hydroxyl radicals was considered [67]. The chosen kinetic model of H_2_O_2_-induced luminol oxidation includes the decomposition of hydrogen peroxide (2), the interaction of luminol with hydroxyl radicals (3), and the final stage of chemiluminescence (4), where P is the reaction product:

(2) $${CeO}_{2}+H_{2}O_{2}\to 2OH\cdot$$

(3) Lum+OH·→Lum∗

(4) Lum∗+Lum∗→P+hυ

As can be seen from Figure 7b, the theoretical kinetic curves fit the experimental data well. The rate constants of reactions (2)–(4) obtained as a result of the mathematical modelling are shown in Table 3.

Since the chemiluminescent analysis technique is based on the registration of the luminescence of the reaction product (4), the constant k_4_ was chosen as the key kinetic parameter used to compare the peroxidase-like activity of cerium dioxide with different particle sizes. The rate of the chemiluminescence reaction of luminol (4) increased by a factor of 1.2 with a two-fold increase in the size of cerium dioxide particles (see Table 3).

It is known that cerium compounds have a high chemical affinity with phosphate ions, which leads to a change in the composition of CeO_2_ surface in phosphate-containing media [68,69,70]. The formation of surface complexes with phosphate species affects the enzyme-like activity of cerium dioxide. Thus, it was previously shown that ageing CeO_2_ in a phosphate-buffered solution reduces the superoxide dismutase activity of cerium dioxide (analysis in Tris-HCl, with pH of 7.4–7.5) [71,72]. Zhao et al. found that phosphate-containing compounds increase the oxidase-like activity of CeO_2_ by up to six times (acetate buffer, with pH of 4) [40].

**Figure 8:**
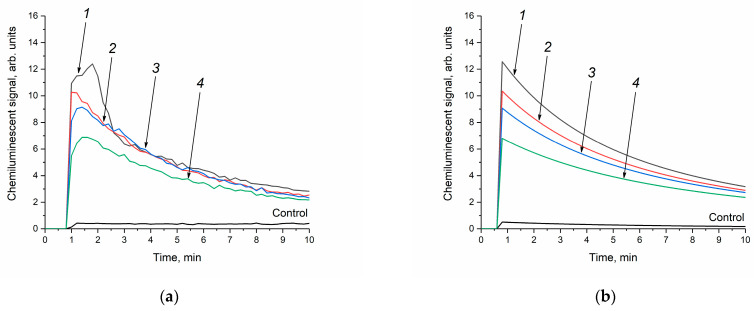
Luminol (50 μM) chemiluminescence kinetic curves in the presence of H_2_O_2_ (10 mM) and CeO_2_ sols (250 μM) in a Tris-HCl buffer solution (100 mM, pH = 7.4): the experimental data were obtained via direct chemiluminescent measurements (**a**), and the calculated curves were obtained through kinetic modelling of the experimental data (**b**). The aqueous ceria sols were obtained from (1) 0.092 M, (2) 0.185 M, (3) 0.277 M and (4) 0.370 M solutions of (NH_4_)_2_[Ce(NO_3_)_6_]. Control: luminol and H_2_O_2_ in a Tris-HCl buffer solution without CeO_2_. The chemiluminescence signal was recorded at 36 °C.

In order to exclude the effect of phosphate species on the enzyme-like activity of cerium dioxide, an analysis was also performed on the peroxidase-like activity of CeO_2_ in Tris-HCl, which has a pH identical to the phosphate-buffered solution (pH = 7.4). Previously, it was shown that the use of Tris-HCl as a dispersion medium does not affect the antioxidant properties of the dispersed CeO_2_ phase [36]. The concentration of cerium dioxide in Tris-HCl was similar to that in the phosphate buffer and amounted to 250 μM. Upon the introduction of the ceria sols into the buffer solutions containing luminol and hydrogen peroxide, the resulting mixtures remained stable with no visible signs of sedimentation. Figure 8a shows that the addition of the CeO_2_ aqueous sols to a mixture of Tris-HCl, hydrogen peroxide and luminol leads to an increase in chemiluminescence intensity, compared with a control without cerium dioxide. At the same time, the luminescence intensity of the luminol oxidation product decreases with increasing CeO_2_ particle size.

The appearance of the chemiluminescence curves in the Tris-HCl solution is almost similar to the shape of the curves recorded in the phosphate-buffered solution (Figure 7a and Figure 8a). However, in the Tris-HCl solution, the chemiluminescence intensity decreases more slowly, indicating a decrease in the rate of reactions (3) and (4). As it can be seen from Table 4, the k_4_ values for the experiments conducted in the Tris-HCl solution are lower than in the phosphate-buffered solution. At the same time, in the Tris-HCl solution, the k_2_ values are three orders of magnitude higher than in the phosphate buffer. The latter observation supports the abovementioned crucial influence of phosphate species on the reactivity of CeO_2_ nanoparticles. In the absence of phosphate species, the rate of CeO_2_ interaction with hydrogen peroxide is significantly higher.

**Figure 9:**
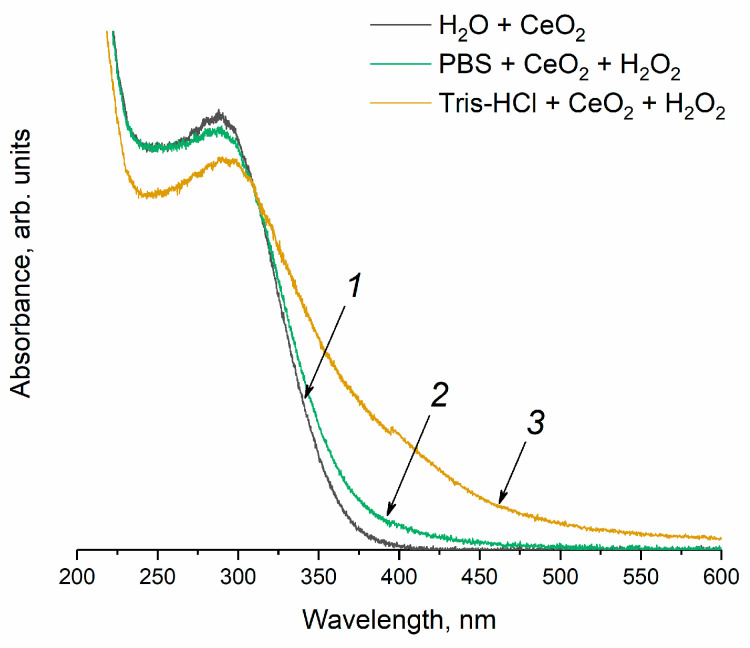
UV-vis absorption spectra of (1) CeO_2_ sol (250 μM, particle size is 2.8 nm) in (2) phosphate-buffered solution and (3) Tris-HCl buffer solution after the addition of hydrogen peroxide (10 mM).

It is common knowledge that the treatment of cerium dioxide sol with hydrogen peroxide can lead to the formation of peroxide and hydroperoxide species on the surface of CeO_2_ [73,74]. When H_2_O_2_ is added to the CeO_2_ sol in the Tris-HCl buffer solution (Figure 9, curve 3), a red shift of the Ce^4+^ absorption band edge (curve 1) is observed, which can be attributed to the formation of peroxo complexes on the CeO_2_ surface [75,76]. The appearance of a shoulder in the absorption spectrum at 400 nm is consistent with the fact that the colloidal solution acquires a yellow colour, which is characteristic of cerium peroxo or hydroperoxo complexes [77,78]. Conversely, the solution of CeO_2_ in the phosphate buffer remains colourless after hydrogen peroxide has been added (curve 2). In this case, the red shift of the absorption band upon treatment with H_2_O_2_ is less pronounced than for the CeO_2_ solution in Tris-HCl, and no shoulder is observed for the absorption spectrum at ~400 nm. This result clearly indicates the difference in the manifestation of the enzyme-like activity of cerium dioxide in a decomposition reaction of hydrogen peroxide in a phosphate-buffered solution and in Tris-HCl.

**Figure 10:**
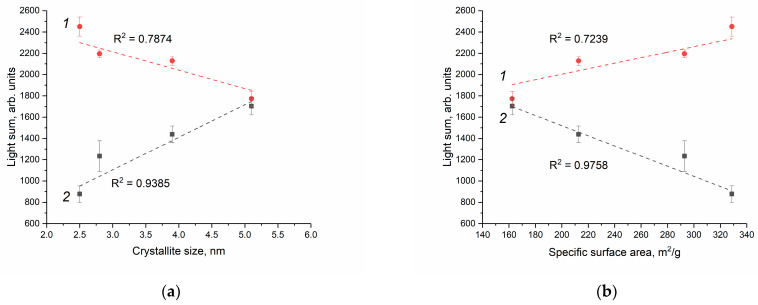
Dependence of light sums for the reaction of luminol oxidation in the presence of cerium dioxide, obtained by integrating the chemiluminescence kinetic curves, on (**a**) particle size and (**b**) CeO_2_ specific surface area in the Tris-HCl buffer solution (1) and phosphate-buffered solution (2). The pH value of both buffer solutions is 7.4.

For the quantitative comparison of the enzyme-like activity of the cerium dioxide sols in the phosphate-buffered solution and Tris-HCl buffer solution, the value of the integrated intensity (light sum) for a selected period of time (10 min) was used. Figure 10 shows the light sum as a function of (a) CeO_2_ particle size and (b) the calculated specific surface area of cerium dioxide. In the phosphate-buffered solution, the peroxidase-like activity of CeO_2_ almost doubles as the particle size doubles, while in the Tris-HCl solution, it decreases by a factor of 1.4.

As follows from Figure 10, the quantitative dependence obtained for the enzyme-like activity of cerium dioxide towards H_2_O_2_ is opposite. In the phosphate-buffered solution, the peroxidase-like activity of CeO_2_ sols increases with an increase in the particle size of the dispersed phase. This result is quite unexpected, since, with a decrease in particle size, the specific surface area available for interaction with the components of the reaction mixture, including reactive oxygen species, increases. Liu et al., however, previously reported a similar result for citrate-stabilised CeO_2_ sols. The peroxidase-like activity of cerium dioxide in an acetate buffer solution (pH 4.5) increased by a factor of ~2.3 with an increase in the particle size by a factor of ~1.5 (from 3 to 4 nm) [79]. It is important to note that the redox activity of cerium dioxide depends on pH and that, according to some data, the prooxidant activity of CeO_2_ increases notably in an acidic environment [37,80].

The interaction of cerium dioxide with the components of the reaction mixture, including phosphate ions, is expected to change the chemical composition of the surface of CeO_2_ particles. The main functional groups on the surface of cerium dioxide are hydroxyl groups, the amount and reactivity of which directly depend on particle size [81,82]. Previously, Wang et al. showed that surface hydroxylation of ceria nanoparticles is a key factor in determining the adsorption kinetics of phosphate species from KH_2_PO_4_ solution. With an increase in the concentration of hydroxyl groups by 1.3 times (from 60 to 76%), the number of adsorbed phosphate species increases by 2.5 times, and the rate of their adsorption increases by 620 times [83]. Thus, phosphate ions can inhibit the interaction of surface-active centres of CeO_2_ with substrates (luminol and hydrogen peroxide). This effect is apparently the main reason for the decrease in the peroxidase-like activity of CeO_2_ with a decrease in particle size from 5.1 to 2.8 nm (Figure 10).

It should be noted that the enzyme-like activity of cerium dioxide with a particle size of 5.1 nm is virtually the same in both the phosphate-buffered solution and the Tris-HCl solution (Figure 10). Conversely, the peroxidase-like activity of CeO_2_ with a particle size of 2.5–3.9 nm in the Tris-HCl solution noticeably exceeds the activity of cerium dioxide in the phosphate-buffered solution. Most probably, this difference can be attributed to the different chemical composition of the Tris-HCl solution and the phosphate-buffered solution. McCormack et al. found that the surface of cerium dioxide particles acquires a negative charge due to the interaction with phosphate species [84]. The surface charge significantly affects the rate of reactions involving charged substrates [34,85]. In the process of free-radical oxidation of luminol, negatively charged intermediates (luminol hydroperoxide anion) can also be formed [58,86]. It is likely that in the phosphate-buffered solution, the interaction of cerium dioxide with negatively charged luminol radical is hindered, resulting in reduced peroxidase-like activity (Figure 10). In addition, the chemiluminescence intensity of luminol depends on the chemical composition of the buffer solution and increases in the presence of halide ions, including Cl^−^ [87].

However, in the Tris-HCl solution, the enzyme-like activity of CeO_2_ towards hydrogen peroxide naturally decreases with increasing particle size (Figure 10). Similarly, Baldim et al. showed that, in a buffer solution of Tris-HCl (pH 7.5), the superoxide dismutase-like activity of colloidal solutions of CeO_2_ decreased by 5 times with an increase in the particle size of cerium dioxide from 5 to 23 nm [76]. Lee et al. found that the antioxidant activity of CeO_2_ stabilised with polyacrylic acid and octylamine also depends on the particle size and decreases by 6 times with the increase in particle size from 4 to 8 nm [88]. In both cases, the decrease in the enzyme-like activity of CeO_2_ was attributed to a decrease in the ratio of Ce^3+^ ions; however, the actual oxidation state of cerium in nanocrystalline CeO_2_ is currently the subject of extensive debates [89]. Recently, the presence of trivalent cerium in nanoscale CeO_2_ has been questioned [81,89].

Since peroxidases and nanozymes that mimic their activity use hydrogen peroxide to oxidise the substrate (including luminol) [90], the sorption of hydrogen peroxide on the CeO_2_ surface can be considered the most important stage of the catalytic reaction. Using computational methods, Wang et al. proposed the following mechanism for the peroxidase-like activity of cerium dioxide: adsorption of hydrogen peroxide, dissociation of H_2_O_2_ with the formation of hydroxyl radicals, and ·OH reduction [91]. This mechanism is fully consistent with the abovementioned reactions (2)–(4) used to describe the kinetics of H_2_O_2_-induced luminol oxidation in the presence of cerium dioxide in a Tris-HCl buffer solution. Therefore, with an increase in the specific surface of CeO_2_ (Table 2) in the absence of phosphate ions, the sorption of hydrogen peroxide increases, and the process of luminol oxidation proceeds more intensively (see Table 4, constant k_4_), which was observed in the Tris-HCl solution (Figure 10b).

Thus, the data obtained in the present study indicate that the determination of the enzyme-like activity of CeO_2_ is a complex task that requires a detailed analysis of a number of factors. The peroxidase-like activity of cerium dioxide is affected by the pH of the medium [37] and the composition of the particle surface [88]. The reactivity of the luminol chemiluminescent probe directly depends on the pH of the buffer solution and, presumably, can be enhanced by the components of the analysed mixture containing halide species (including the buffer). It is also necessary to take into account the presence of species in the reaction mixture that can specifically interact with the enzyme-like material and significantly change its activity. The correct choice of the chemical environment is fundamentally important for obtaining reliable data on the enzyme-like activity of CeO_2_, including its activity under conditions close to the intracellular environment. An analysis of the interaction of cerium dioxide with reactive oxygen species requires further thorough investigation, and this work gives impetus to the development of appropriate approaches.

## 3. Materials and Methods

### 3.1. Materials

Ammonium cerium(IV) nitrate (99.9%, Lanhit (Moscow, Russia)), isopropyl alcohol (99.9%, Chimmed (Moscow, Russia)), hydrogen peroxide (99.9%, Chimmed), luminol (Sigma-Aldrich, St. Louis, MO, USA, 123072), potassium dihydrogen phosphate (Sigma-Aldrich, P0662), potassium hydrogen phosphate (Sigma-Aldrich, P5655), tris-hydrochloride (Merck, Lowe, NJ, USA, 10812846001), and Milli-Ω Water (18.2 MΩ/cm).

### 3.2. Synthesis of CeO_2_ Sols

Four samples of nanoceria were prepared via thermohydrolysis of ammonium cerium(IV) nitrate without using additives. (NH_4_)_2_[Ce(NO_3_)_6_] solutions with different concentrations (0.092, 0.185, 0.277, 0.370 and 0.554 M) were placed in a 100 mL Synthware^TM^ autoclave (filling degree of 25%) and heated at 95 °C for 24 h. The resulting yellow precipitates were separated by centrifugation (relative centrifugal force of 20,000× *g*), washed three or four times with isopropyl alcohol, re-dispersed in an appropriate amount of distilled water, and boiled for 3 h to remove the remaining isopropanol. The concentration of CeO_2_ in the resulting sols was determined gravimetrically. Before the analysis of enzyme-like activity, the resulting sols were diluted to the same concentration, *c*(CeO_2_) = 0.05 M.

### 3.3. Characterisation Methods

The X-ray powder diffraction pattern analysis (XRD) of the sols (dried at 50 °C) was performed using a Bruker (Billerica, MA, USA) D8 Advance diffractometer (CuKα radiation) in the angle range of 20–80° 2θ, with a step of 0.02° 2θ and a signal acquisition time of 0.4 s per step. The full-profile analysis of diffraction patterns was performed using the Bruker (Billerica, MA, USA) TOPAS v.4.2 software, and the diffraction maxima were fitted to Voigt pseudo-functions.

CeO_2_ nanoparticles were investigated using a JEOL JEM 2100 UHR (Akisima, Tokyo, Japan) transmission electron microscope (TEM) at an acceleration voltage of 200 kV. A drop of the aqueous sol was placed on the formvar/carbon Cu grid (Ted Pella Inc., Redding, CA, USA) and left to evaporate. The samples were treated in an HPT-100 plasma cleaner (Henniker Plasma, Runcorn, UK) prior to being inserted into the microscope chamber in order to remove organic residues from the grid surface. The acquisition of micrographs was performed using a Quemesa 11 MegaPixel CCD (Olympus, Shinjuku/Tokyo, Japan) camera in the bright field mode.

The dynamic light scattering (DLS) and ζ-potential measurements were carried out using a Photocor Compact-Z analyser (Photocor, Moscow, Russia) equipped with a 636.65 nm laser. The sample preparation was carried out using Milli-Ω Water (18.2 MΩ/cm), and a temperature equilibrium was ensured between the sample cell and the cuvette holder. The correlation function for each of the samples was gained by averaging 10 curves, each being acquired for 20 s. Then, the data were filtered by adjusting the permissible deviation of the scattering intensity from the average value (no more than 10%), taking into account the shift of the baseline.

The optical absorption spectra were recorded using quartz cuvettes (10.0 mm optical path length) in a 200–600 nm range, at 0.1 nm steps, on an SF-2000 spectrophotometer (OKB Spectr, Saint-Petersburg, Russia) with a deuterium-halogen light source.

The IR spectra of the samples were recorded in attenuated total reflection geometry using a Spectrum 65 FT IR spectrometer (Perkin Elmer, Waltham, MA, USA) with a spectral resolution of 2 cm^−1^, in the wavenumber range of 400–4000 cm^–1^.

The Raman spectra were recorded using a Confotec NR500 spectrometer (SOL Instruments, Minsk, Belarus) with a 514 nm laser, using a ×40 (NA = 0.75) lens at ~30 mW laser power. The spectral resolution was 0.8 cm^−1^.

The enzyme-like activity (peroxidase/catalase-like) of the CeO_2_ sols was investigated using the chemiluminescent method with the reaction of luminol oxidation in the presence of hydrogen peroxide in a phosphate (100 mM, pH of 7.4, K_2_HPO_4_) or a Tris-HCl (100 mM, pH of 7.4) buffer solution, at 36 °C. The background luminescence was recorded for 60 s after mixing the solutions of hydrogen peroxide (10 mM) and luminol (50 μM). Then, an aliquot (5 μL) of the CeO_2_ sol was added (250 μM), and a chemiluminescent signal was recorded for 10 min. Light sums were calculated via numerical integration of the chemiluminescence curves using the PowerGraph software v.3.3 (Moscow, Russia).

## 4. Conclusions

Thermohydrolysis of solutions of ceric ammonium nitrate with concentrations in the range of 0.092–0.370 M produced ceria colloidal solutions with different particle sizes in the range of 2–5 nm. All the obtained sols showed good colloid stability over three months of storage under ambient conditions. For the quantitative analysis of the enzyme-like activity of the CeO_2_ sols with respect to hydrogen peroxide, a chemiluminescent method was chosen to determine reactive oxygen species based on the interaction of the probe (luminol) with hydroxyl radicals. To address the effect of chemical environment on the CeO_2_ nanozyme property, the analysis was conducted using two different buffers (Tris-HCl and PBS) at an identical pH (7.4). The peroxidase-like activity of cerium dioxide in the Tris-HCl buffer solution decreased with an increase in particle size. The opposite dependence was registered in the phosphate-buffered solution, while the rate of luminol oxidation in the phosphate-rich medium was significantly lower than in the Tris-HCl medium. According to the kinetic modelling, in the phosphate-rich medium, the rate of CeO_2_ interaction with H_2_O_2_ was more than three orders of magnitude lower than in the Tris-HCl medium. The mechanisms of the enzyme-like activity of CeO_2_ nanoparticles in different media are discussed.

## Figures and Tables

**Table 1:** Synthesis conditions and concentrations of aqueous CeO_2_ sols prepared by thermohydrolysis of ceric ammonium nitrate.

Sample	(NH_4_)_2_[Ce(NO_3_)_6_], M	c(CeO_2_), M (g/L)	Yield, %
1	0.092	0.09 (15.6)	85
2	0.185	0.15 (26.1)	89
3	0.277	0.15 (26.2)	90
4	0.370	0.15 (25.9)	86

**Table 2:** The main parameters of CeO_2_ sols.

CeO_2_ Sample	Concentration of the Initial (NH_4_)_2_[Ce(NO_3_)_6_] Aqueous Solution (M)	Particle Size Estimated from XRD Data (D_XRD_, nm)	Particle Size Estimated from DLS Data (D_DLS_, nm)	ζ-Potential (mV)	Specific Surface Area (SSA *, m^2^/g)	Proportion of Surface Cerium Atoms, (Ce_surf_ *, %)
1	0.092	2.5 ± 0.4	10	+29.9 ± 0.3	329	60
2	0.185	2.8 ± 0.3	19	+31.8 ± 0.4	293	54
3	0.277	3.9 ± 0.3	38	+34.4 ± 0.6	213	39
4	0.370	5.1 ± 0.4	48	+38.2 ± 0.7	162	30

* Calculated values.

**Table 3:** Rate constants of H_2_O_2_-induced luminol oxidation reactions in the presence of aqueous sols of cerium dioxide in a phosphate-buffered solution (100 mM, pH = 7.4).

Sample	Particle Size Estimated from XRD Data (D_XRD_, nm)	k_2_ (μM/min)	k_3_ (μM/min)	k_4_ (μM/min)
Control	–	6.9 × 10^4^	1.9 × 10^−6^	3.7 × 10^−5^
1	2.5 ± 0.4	8.9 × 10^4^	2.9 × 10^−6^	4.7 × 10^−5^
2	2.8 ± 0.3	9.7 × 10^4^	3.7 × 10^−6^	5.3 × 10^−5^
3	3.9 ±0.3	9.8 × 10^4^	3.8 × 10^−6^	5.3 × 10^−5^
4	5.1 ± 0.4	1.0 × 10^5^	4.3 × 10^−6^	5.5 × 10^−5^

**Table 4:** Rate constants of H_2_O_2_-induced luminol oxidation in the presence of aqueous cerium dioxide sols in a Tris-HCl solution (100 mM, pH = 7.4).

Sample	Particle Size Estimated from XRD Data (D_XRD_, nm)	k_2_ (μM/min)	k_3_ (μM/min)	k_4_ (μM/min)
Control	–	6.9 × 10^4^	1.5 × 10^−6^	3.0 × 10^−5^
1	2.5 ± 0.4	1.2 × 10^8^	1.6 × 10^−8^	2.0 × 10^−6^
2	2.8 ± 0.3	1.2 × 10^8^	1.6 × 10^−8^	1.8 × 10^−6^
3	3.9 ± 0.3	1.1 × 10^8^	1.6 × 10^−8^	1.7 × 10^−6^
4	5.1 ± 0.4	1.1 × 10^8^	1.5 × 10^−8^	1.5 × 10^−6^

## Data Availability

Data are contained within this article.

## Supplementary Materials

The following supporting information can be downloaded at https://www.mdpi.com/article/10.3390/molecules28093811/s1. Figure S1: TEM image of CeO_2_ nanoparticles in the ceria sol obtained from 0.185 M aqueous solution of (NH_4_)_2_[Ce(NO_3_)_6_]; Figure S2: ζ-potential values of the CeO_2_ aqueous sols prepared from (NH_4_)_2_[Ce(NO_3_)_6_] solutions after three months of storage under ambient conditions.
