# Supplementary material for: Peroxidase-like Activity of CeO2 Nanozymes: Particle Size and Chemical Environment Matter
Source: Molecules. 2023 Apr 29;28(9):3811. doi: 10.3390/molecules28093811 (PMC10180353; doi:10.3390/molecules28093811)
Supplement: Supplementary file 1 [file molecules-28-03811-s001.zip › molecules-2313232-supplementary.pdf]

Supplementary Information

Peroxidase-like Activity of CeO_2 Nanozymes: Particle Size and Chemical Environment Matter

Arina D. Filippova [1], Madina M. Sozarukova [1], Alexander E. Baranchikov [1], Sergey Yu. Kottsov [1], Kirill A. Cherednichenko [2] and Vladimir K. Ivanov [1,*]

[1] Kurnakov Institute of General and Inorganic Chemistry of the Russian Academy of Sciences, 119991 Moscow, Russia
[2] Department of Physical and Colloid Chemistry, Faculty of Chemical and Environmental Engineering, National University of Oil and Gas "Gubkin University", 119991 Moscow, Russia
* Correspondence: van@igic.ras.ru

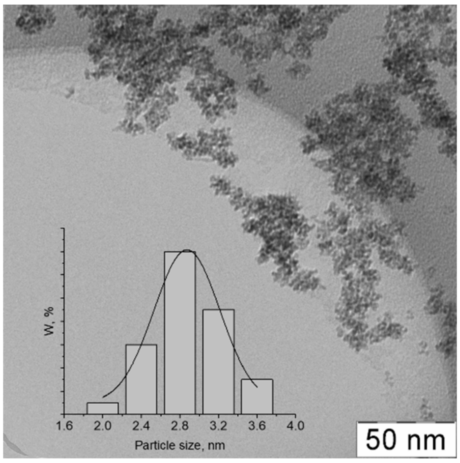

Figure S1. TEM image of CeO_2 nanoparticles in ceria sol obtained from 0.185 M aqueous solution of $(NH_4)_2[Ce(NO_3)_6]$.

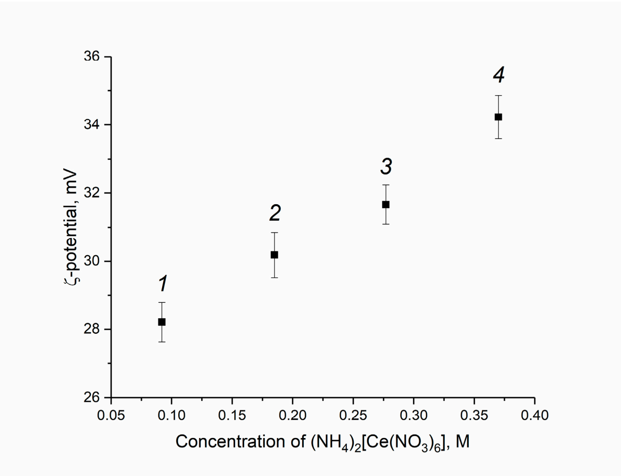

Figure S2. ζ-potential values of CeO_2 aqueous sols prepared from $(NH_4)_2[Ce(NO_3)_6]$ solutions after three months of storage under ambient conditions. Sols were obtained from (1) 0.092 M, (2) 0.185 M, (3) 0.370 M, (4) 0.554 M solutions of $(NH_4)_2[Ce(NO_3)_6]$.
